# Disparities in Cardiovascular Prosthetic Device-Related Deaths: A Longitudinal Mortality Analysis in the United States

**DOI:** 10.1016/j.jacadv.2025.101595

**Published:** 2025-02-04

**Authors:** Hoang Nhat Pham, Ramzi Ibrahim, Mahmoud Abdelnabi, Mohammed Salih, Mohammed Y. Khanji, C. Anwar A. Chahal, Mamas A. Mamas, Justin Lee, Kwan Lee

**Affiliations:** a Department of Medicine, University of Arizona Tucson, Tucson, Arizona, USA; b Department of Cardiovascular Medicine, Mayo Clinic, Phoenix, Arizona, USA; c Heart Hospital–Baylor University Medical Center, Plano, Texas, USA; d Newham University Hospital and Barts Heart Centre, London, UK; e William Harvey Research Institute, Queen Mary University of London, London, United Kingdom; f Center for Inherited Cardiovascular Diseases, WellSpan Health, York, Pennsylvania, USA; g Department of Cardiology, Barts Heart Centre, London, United Kingdom; h Department of Cardiovascular Medicine, Mayo Clinic, Rochester, Minnesota, USA; i Keele Cardiovascular Research Group, Keele University, Keele, United Kingdom; j Department of Cardiovascular Medicine, Cleveland Clinic, Cleveland, Ohio, USA

**Keywords:** cardiovascular prosthetic device, CDC WONDER, mortality disparities

**What is the clinical question being addressed?**

What are the trends and disparities in mortality associated with complications of cardiovascular prosthetic devices (CVPDs)?

**What is the main finding?**

From 1999 to 2020, mortality disparities related to CVPD complications vary by complications type, sex, race/ethnicity, and region. Infection-related mortality is approximately 5.4 times higher than mechanical-related mortality, with a slower decline over time.

CVPDs have undergone substantial development in recent years. Despite improvements in design and surgical procedures, adverse events associated with CVPDs are expected to increase as interventions become more frequent.^1^ Limited data exist on mortality related to CVPD complications. We investigated the longitudinal trends and disparities in mechanical and infection-related CVPD mortality.

Mortality and demographic data were acquired from the CDC Wide-ranging Online Data for Epidemiologic Research database. Deaths associated with International Classification of Diseases-10th Revision (ICD-10) codes related to mechanical (T82.0-T82.5) and infective (T82.6-T82.7) complications of CVPDs were identified from multiple cause-of-death records from 1999 to 2020. CVPDs, as defined by ICD-10 codes, include prosthetic heart valve, cardiac electronic devices, and other cardiac and vascular devices and implants. Using the direct method of standardization, we estimated age-adjusted mortality rates (AAMRs) per 1,000,000 population (standardized to the year 2000 population). Analysis of temporal trends was evaluated using log-linear regression models, particularly through fitting trends in the smallest number of significant joinpoints (Joinpoint Regression-National Cancer Institute). Monte-Carlo permutation tests were used to estimate the average annual percentage change (AAPC) from 1999 until 2020. The study was exempt from Institutional Review Board approval given the publicly available and anonymized nature of the data.

Between 1999 and 2020, a total of 33,932 CVPD-related deaths were identified, with 28,844 deaths attributed to infection and 5,201 deaths attributed to mechanical issues. Mortality was higher related to infective (AAMR 3.95 [95% CI: 3.90-4.00]) than mechanical (AAMR 0.72 [95% CI: 0.70-0.74]) complications. Despite the decreasing trends observed in both causes of death, mechanical-related mortality (AAPC −4.54%; 95% CI: −6.50 to −2.54; *P* < 0.001]) experienced a more rapid decline, though statistically insignificant, compared to infection-related mortality (AAPC −1.98%; 95% CI: −2.98 to −0.98; *P* < 0.001) (Figure 1). Compared with females, males had higher total AAMR related to mechanical (AAMR 0.85 [95% CI: 0.82-0.89] vs 0.62 [95% CI: 0.59-0.64]) and infective complications (AAMR 5.05 [95% CI: 4.97-5.13] vs 3.10 [95% CI: 3.05-3.16]). Mortality was higher in rural regions compared to urban for both mechanical-related (AAMR 0.92 [95% CI: 0.87-0.98] vs 0.68 [95% CI: 0.66-0.71], respectively) and infection-related (AAMR 4.23 [95% CI: 4.12-4.35] vs 3.89 [95% CI: 3.84-3.94] respectively) deaths. Compared to Hispanic populations, non-Hispanic populations had a higher AAMR for both mechanical (AAMR 0.74 [95% CI: 0.72-0.76] vs 0.50 [95% CI: 0.44-0.56], respectively) and infection-related (AAMR 4.00 [95% CI: 3.95-4.05] vs 3.22 [95% CI: 3.07-3.37], respectively) deaths. Non- Hispanic Black decedents had the highest AAMR for both mechanical-related (AAMR 1.16 [95% CI: 1.08-1.24]) and infection-related (AAMR 7.34 [95% CI: 7.14-7.54]) deaths compared to other racial populations. Mechanical-related mortality was highest in the Midwestern U.S. regions (AAMR 0.79 [95% CI: 0.75-0.84]) while infection-related mortality was highest in the Southern U.S. regions (AAMR 4.20 [95% CI: 4.12-4.28]).

**Figure 1:**
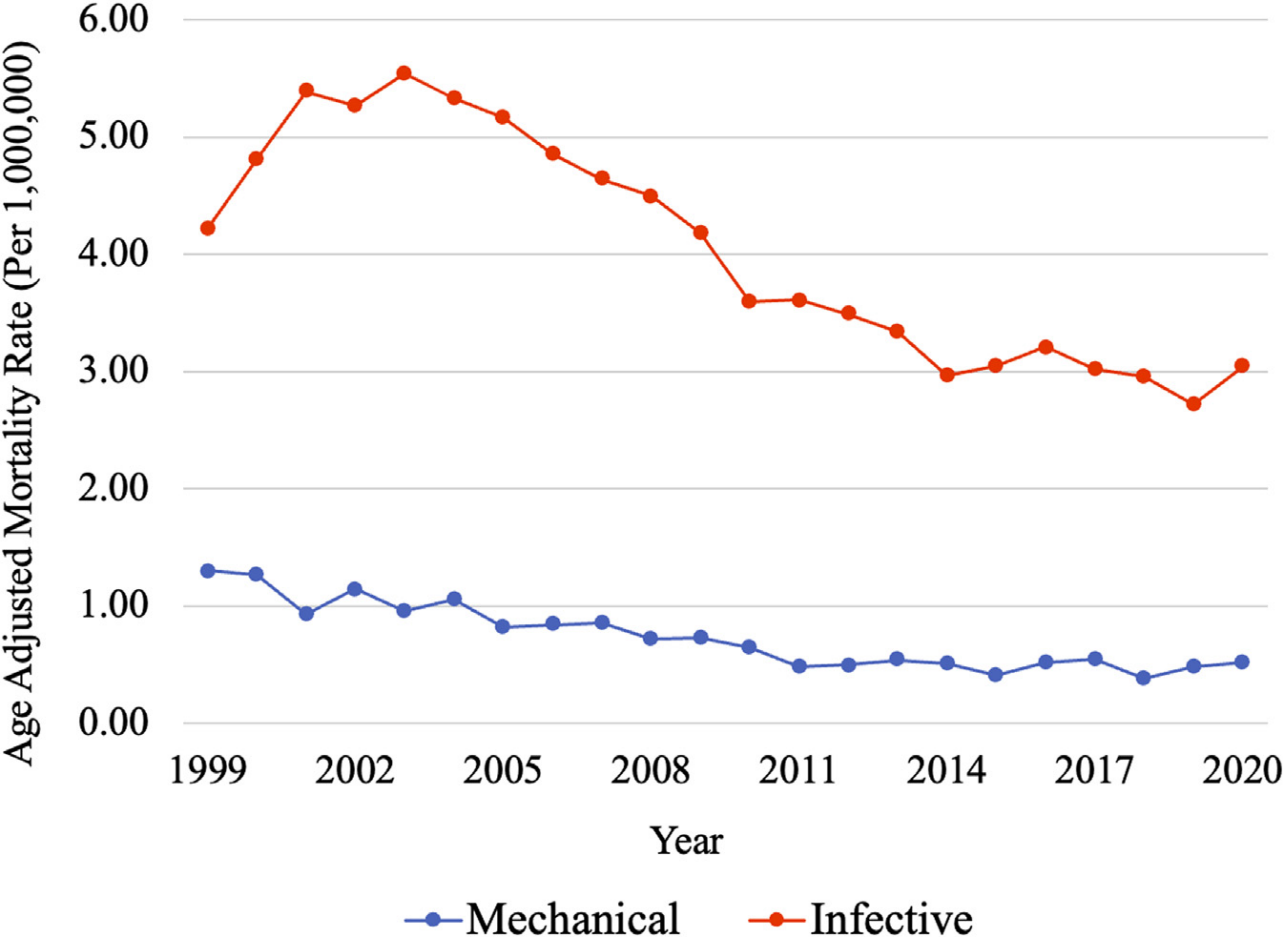
**Cardiovascular Prosthetic Device Mortality Trends** Trends in cardiovascular prosthetic device-related mortality from 1999 to 2020, stratified by mechanical- and infective-related complications.

Our analysis revealed infection- and mechanical-related CVPD mortality differences among different population groups. Infection-related mortality rate was approximately 5.4 times higher with a slower decline compared to mechanical-related mortality. Higher mortality related to infective complications is likely multifactorial, including lack of preventive measures prior to nondental procedures, and rising prevalence of CVPDs in elderly population with severe comorbidities.^2^ In contrast, technological advances in CVPD design and operative techniques with increased accessibility to safer anticoagulation agents has led to significantly reduced mechanical complications.^3^ Additionally, the remarkable increase in the use of transcatheter valve placement in recent years may have contributed to the reduction in both mechanical and infection-related mortality.^4^

Disproportionate CVPD-related mortality between rural-urban could be explained by varying access to health care, poor health literacy, and shortage of specialists in rural regions, leading to missed opportunities for treatment.^5^ The shortage of cardiac specialists in rural areas has resulted in general cardiologists performing these procedures which might result in increased workload and higher complications rates.^1^ In contrast, urban tertiary hospitals with advanced equipment and higher procedure volumes experience fewer adverse outcomes and lower mortality rates.^1^ The higher mortality rate among Black populations can be driven by several interrelated factors, including higher comorbidity burden adversely affecting outcome of cardiovascular procedures, lower socioeconomic status, structural racism, and behavioral culture that limit access to quality health care and result in inadequate postoperative care and follow-up.^6^ Interestingly, our analysis showed lower mortality in the Hispanic population, which has been previously reported as the “Hispanic paradox,” potentially due to their stronger social support networks and healthier lifestyle behaviors.^7^

This analysis is limited by ICD-10 code misclassification, potential ecological fallacy, the inability to distinguish between different types of CVPDs, the absence of individual-level data and time intervals between CVPD procedures and adverse outcomes, which can hinder the identification of important confounding factors. Despite these limitations requiring cautious interpretation of our results, the strength of our analysis lies in the utilization of nationally representative sample, a reliable source to assess mortality trends and disparities of CVPD complications.

Our results revealed mortality disparities related to mechanical and infective CVPD complications across sexes, races and ethnicities, and geographical regions. Further investigation is imperative to identify contributing factors to these disparities to enable more equitable care improve outcomes.

## Funding support and author disclosures

The authors have reported that they have no relationships relevant to the contents of this paper to disclose.
